# *In situ* co-deposition synthesis for collagen-Astragalus polysaccharide composite with intrafibrillar mineralization as potential biomimetic-bone repair materials

**DOI:** 10.1093/rb/rbae070

**Published:** 2024-06-21

**Authors:** Han Li, Ziying Guan, Liren Wei, Jian Lu, Yanfei Tan, Qingrong Wei

**Affiliations:** National Engineering Research Center for Biomaterials (NERCB), Sichuan University, Chengdu 610065, P.R. China; College of Biomedical Engineering, Sichuan University, Chengdu 610065, P.R. China; National Engineering Research Center for Biomaterials (NERCB), Sichuan University, Chengdu 610065, P.R. China; College of Biomedical Engineering, Sichuan University, Chengdu 610065, P.R. China; National Engineering Research Center for Biomaterials (NERCB), Sichuan University, Chengdu 610065, P.R. China; College of Biomedical Engineering, Sichuan University, Chengdu 610065, P.R. China; National Engineering Research Center for Biomaterials (NERCB), Sichuan University, Chengdu 610065, P.R. China; College of Biomedical Engineering, Sichuan University, Chengdu 610065, P.R. China; National Engineering Research Center for Biomaterials (NERCB), Sichuan University, Chengdu 610065, P.R. China; College of Biomedical Engineering, Sichuan University, Chengdu 610065, P.R. China; National Engineering Research Center for Biomaterials (NERCB), Sichuan University, Chengdu 610065, P.R. China; College of Biomedical Engineering, Sichuan University, Chengdu 610065, P.R. China

**Keywords:** intrafibrillar mineralization, collagen fibril, co-deposition, Astragalus polysaccharide, biomimetic-bone repair

## Abstract

A hybrid material possessing both componential and structural imitation of bone tissue is the preferable composites for bone defect repair. Inspired by the microarchitecture of native bone, this work synthesized *in vitro* a functional mineralized collagen fibril (MCF) material by utilizing the method of *in situ* co-precipitation, which was designed to proceed in the presence of Astragalus polysaccharide (APS), thus achieving APS load within the biomineralized collagen-Astragalus polysaccharide (MCAPS) fibrils. Transmission electron microscope (TEM), selected area electron diffraction (SAED) and scanning electronic microscopy (SEM) identified the details of the intrafibrillar mineralization of the MCAPS fibrils, almost mimicking the secondary level of bone tissue microstructure. A relatively uniform and continuous mineral layer formed on and within all collagen fibrils and the mineral phase was identified as typical weak-crystalline hydroxyapatite (HA) with a Ca/P ratio of about 1.53. The proliferation of bone marrow-derived mesenchymal stem cells (BMSC) and mouse embryo osteoblast precursor cells (MC3T3-E1) obtained a significant promotion by MCAPS. As for the osteogenic properties of MCAPS, a distinct increase in the alkaline phosphatase (ALP) activity and the number of calcium nodules (CN) in BMSC and MC3T3-E1 was detected. The up-regulation of three osteogenic-related genes of RUNX-2, BMP-2 and OCN were confirmed via reverse transcription-quantitative polymerase chain reaction (RT-qPCR) to further verify the osteogenic performance promotion of MCAPS. A period of 14 days of culture demonstrated that MCAPS-L exhibited a preferable efficacy in enhancing ALP activity and CN quantity, as well as in promoting the expression of osteogenic-related genes over MCAPS-M and MCAPS-H, indicating that a lower dose of APS within the material of MCAPS is more appropriate for its osteogenesis promotion properties.

## Graphical Abstract

**Figure:**
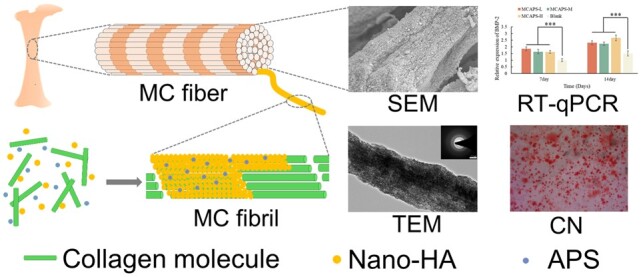

## Introduction

Each year, over 2.2 million bone graft surgeries are performed in orthopedics, stomatology and neurosurgery [1–3]. The materials needed clinically for bone defect repair include autologous bone, allogeneic bone and artificial bone [2]. Up to present, artificial bone materials have developed from initial inert fillers to osteoinductive repair materials with bioactivity, especially biomimetic materials with multi-component [1, 4, 5]. In the 1990s, the concept of bone tissue engineering was proposed, which involves suitable scaffold materials, seed cells and cell growth factors [5, 6]. Scaffold materials include the type of metal, polymer, inorganic and composite materials according to their component quality [5–7]. Based on the respective characteristics of metal, polymer and inorganic materials, the composite materials comprising organic and inorganic components learn from each other in terms of their advantageous performances, which not only have the strength and bioactivity offered by inorganic part but also have the toughness and excellent biocompatibility coming from organic component [8, 9].

Bone tissue is a typical composite material composed of organic and inorganic components, which mainly are minerals of carbonated hydroxyapatite (HA) nanocrystals (ca. 65%), and organic matrix type I collagen (ca. 34%) [10–12]. Other components are non-collagenous proteins and water [13, 14]. From macroscopic to microscopic angles, the structures of bone tissue can be broadly classified into seven levels: bone tissue, cancellous and dense bone, bone units, layers of mineralized collagen fibrils (MCFs) arranged in parallel and staggered rows, bundles of MCF, hydroxyapatite nanocrystals and collagen macromolecules [10, 12, 14, 15].

MCF are the basic constituent units of bone tissue and belong to the secondary level of bone microstructures [11, 16, 17]. A great deal of efforts has been exerted in the development of mineralized collagen fibrillar materials by *in vitro* synthesis for bone tissue repair [18–21]. The initial method was a componential imitation by simply mixing collagen fibrils with hydroxyapatite nanoparticles [22–24]. However, this method cannot replicate the well-distributed and ordered combination between collagen fibril and nanohydroxyapatite of native bone tissue [25]. Then immersion of collagen sponges in simulated body fluid (SBF) results in the nucleation of HA occurring heterogeneously on collagen fibrils by conventional crystallization reaction [26, 27]. While this type of method has fabricated simple composites with a surface layer of random clusters of HA on the surface of collagen fibrils, they have failed to achieve biomineralization within collagen fibrillar microstructure, which is not genuine intrafibrillar mineralization [26, 27].

On the other hand, to further enhance the functionality of HA-collagen scaffolds, osteogenesis-related growth factors have usually been loaded or grafted onto these scaffold materials for optimized performances [7, 28, 29], such as vascular endothelial growth factor (VEGF), bone morphogenetic protein-2 (BMP-2), and transforming growth factor-β2 (TGF-β2) [7, 28, 29]. However, growth factors generally are short half-life, unstable release, high cost and rapid degradation. Consequently, a need for development of cost-effective, reliable and safe alternatives is necessary.

Traditional Chinese medicines have been applied clinically in China for thousands of years, and their efficacy, reliability and safety have been fully proven over a long period of practice history [30–32]. Basing on the classical theory and clinical applications of traditional Chinese medicines, a variety of Chinese medicines have a quite positive effect on the regeneration of bone tissue, deriving from their efficacy of ‘tonifying the kidneys and strengthening the bones’ [33–35]. These representative traditional Chinese medicines include Epimedium, Rhizoma Drynariae, Eucommia, Astragalus, etc. [32, 36]. Such medicines can promote the proliferation and differentiation of osteoblasts and inhibit the activity of osteoclasts, which is conducive to bone tissue regeneration [32, 36]. Compared with growth factor substances, traditional Chinese medicine ingredients are widely available, inexpensive, safe and reliable and pollution-free [32]. It is of great significance to combine the bioactive ingredients of such traditional Chinese medicines with the scaffold of bone tissue engineering to construct a more functional scaffold material with some better qualities to regulate osteoblast behavior for bone defect repair [32–36]. However, most of the active ingredients of traditional Chinese medicines, such as icariin, quercetin, naringenin, etc., have poor water solubility and need organic solvents for further utilization, which limits their convenient applications [32, 35–37]. Astragalus polysaccharide (APS), a main efficacy component of Astragalus, not only being antiviral, anti-aging, and antioxidant, but also has the capability of facilitating bone regeneration by promoting the proliferation and differentiation of osteoblasts [38–40]. Simultaneously, APS can enhance the synthesis of type II collagen in chondrocytes, which is crucial in preserving the shape and function of cartilage [38–40].

Therefore, from a biomimetic perspective, this work is designed to synthesize *in vitro* the functional biomineralized composite collagen fibrils with a similar structure to the secondary level of bone tissue microstructure. In this synthesis route, the self-assembly behavior of collagen macromolecules occurred simultaneously with the mineralization initiation. That is, while the collagen macromolecules self-assembled into fibrils, the formation of mineral nuclei also happened by calcium and phosphate ions aggregating inside and on the surface of the collagen fibrils, resulting in uniform and intrafibrillar mineralization of collagen fibrils, which is the closest biomimetic mineralization to the bone tissue microstructures [21, 41]. Of greater significance, we introduced water-soluble APS into the synthesis procedure of these biomineralized collagen fibrils, participating in their complete growth process, thus obtaining a novel kind of biomineralized collagen-Astragalus polysaccharide (MCAPS) composite fibrils as an innovative potential bone repair material.

## Materials and methods

### Materials

High-purity medical collagen (type I) was extracted from calfskin in our laboratory. Anhydrous CaCl_2_, Na_2_HPO_4_ (Chengdu Cologne Chemical Co, Ltd) and APS (Beijing Solarbio Science & Technology Co, Ltd) were used as received state without further disposing. Bone marrow-derived mesenchymal stem cell (BMSC) rat and MC3T3-E1 were purchased from National Collection of Authenticated Cell Cultures. Cell culture dishes and cell well plates for cell culture were provided by Corning Incorporated. The α-mem medium, fetal bovine serum, penicillin, streptomycin and trypsin were purchased from Gibco Incorporated.

### Synthesis of biomineralized collagen-Astragalus polysaccharide composite fibrils

The highly purified soluble collagen was dissolved in hydrochloric acid (pH 2.5) to obtain 70 ml acidic collagen solution at a 3-mg/ml concentration. Dropwise addition of 10 ml CaCl_2_ solution (1.0 mol/l) and APS solution into the collagen solution were performed in sequence with moderately stirring, which was followed by the dropwise addition of 10 ml Na_2_HPO_4_ solution (0.6 mol/l). The pH of this composite system was then gradually adjusted to 9 with NaOH solution under gently stirring. And the system was left to stand for 72 h at room temperature. Afterwards, the precipitate was separated from the suspension and washed with deionized water several times and transferred to a mold for freeze-drying. Then the freeze-dried sponge was thoroughly grinded to obtain the powder of MCAPS. The samples were labeled as MCAPS-L, MCAPS-M and MCAPS-H according to the final concentration of APSs (0.25 mg/ml, 0.5 mg/ml, 1.0 mg/ml) in the biomineralized composite fibrils reaction system, and the sample of MCF without APS was applied as a control group and labelled as Control.

### Analyses of infrared spectroscopy

The sample and potassium bromide powder were mixed at a mass ratio of 1:100 in a pellet mold, and then pressed into circular slices, which were scanned under an infrared (IR) spectrometer (INVENIO R, Bruker, Germany) with a scanning wavelength range of 4000–400 cm^−1^.

### X-ray diffraction

The phase characterization of the mineralized composite fibrils was performed on an X-ray single crystal diffractometer (XRD, Xcalibur E, Oxford, United Kingdom) using monochromator Cu K_α_ radiation at 40 kV and 40 mA with scanning at 0.03° steps from 20 to 60° (2*θ*).

### Scanning electronic microscopy and energy-dispersive spectroscopy

The micromorphology of the mineralized composite collagen fibrils was analyzed using a field-emission scanning electron microscope (SEM, S-4800, Hitachi, Japan). The specimens were fixed on conductive adhesive for Au sputter-coating. The major element distribution analyses of the specimens without Au sputter-coating were performed with energy-dispersive spectroscopy (EDS, ProX, Phenom, Netherlands).

### Transmission electron microscope and selected area electron diffraction

The fine composite collagen fibrils were fully dispersed in anhydrous ethanol to form suspensions, which were absorbed by a capillary tube and released on a copper grid for drying. Then the fibrils-loaded copper grid was placed under a transmission electron microscope (TEM, Tecnai G2 F20 S-TWIN, FEI, USA) to explore the micro-architecture of the mineralized composite collagen fibrils. And the crystal type and crystal orientation of minerals inside and outside collagen fibrils were observed by SAED.

### Release of APS *in vitro* from the composite fibrils of MCAPS

The composite fibrils of MCAPS were soaked in phosphate buffer saline (PBS) at room temperature, and the supernatant was sampled at Days 1, 3, 5, 8, 14 and 21, respectively, for determining the content of the APS released in the supernatant by a UV spectrophotometer (U-2910, Hitachi, Japan). MCAPS was then dissolved in dilute hydrochloric acid and the supernatant was collected to determine the content of APS in MCAPS.

### Thermogravimetric analysis

The thermogravimetric analysis (TGA) of the composite fibrils of MCAPS was conducted under a thermogravimetric analyzer (TGA/DSC2, Mettler-Toledo, Switzerland) in nitrogen atmosphere with a rate of 10°C/min from room temperature to 1000°C.

### Cell culture

BMSC and MC3T3-E1 cells, cultured in α-MEM medium supplemented with 10% fetal bovine serum and 1% penicillin-streptomycin, were incubated in 5% CO_2_ humidified atmosphere at 37°C, and the medium was changed every 2 days.

### Cell proliferation

The extractive was obtained by co-incubating the materials of MCAPS-L, MCAPS-M, MCAPS-H and Control, respectively with the medium at constant temperature under shaking. BMSCs and MC3T3-E1 were seeded at a density of 5 × 10^3^ in 48-well plates, respectively. The experimental groups received the extractive, while the Blank group received only the medium without the extractive. All groups were cultured under the same conditions for 1, 3 and 7 days. At each time point, CCK-8 reagent was added to every target well with incubation for 1 h. Then the supernatant was collected from the wells for reading their absorbance at 450 nm on a microplate reader (Varioskan Flash, Thermo Fisher Scientific, USA).

### Live/dead staining

Also, at a concentration of 5 × 10^3^ cells per well in 48-well plates, BMSC and MC3T3-E1 cells were inoculated, respectively. The experiment groups were treated with the MCAPS extractive, while the Blank group was treated only with the blank medium. After incubation of 3 and 7 days, at each of the time points, the cells were washed with PBS and stained for 15 min with Calcein/PI double staining kit. After discarding the staining solution, the plates were placed under an inverted fluorescence microscope (IX73, Olympus, Japan) for observation and photography.

### Quantification assay of alkaline phosphatase

After respective immersion in different extractive solutions from a series of the materials of MCAPS, BMSC and MC3T3-E1 cells were seeded at a density of 8 × 10^3^ per well in 24-well plates and cultured for 7 and 14 days, which was followed by fixing with 4% paraformaldehyde for 15 min, washing in PBS, and then staining with ALP staining-reagent for observation under an inverted microscope. Additionally, at Day 7 and Day 14, the cells were added with cell lysis buffer after being washed in PBS and the ALP content was detected via an ALP quantitative assay kit.

### Alizarin Red staining

BMSC and MC3T3-E1 cells were seeded at a density of 2 × 10^4^ per well in a 6-well plate, respectively, and the cells were cultured in the medium containing the extractive and osteogenic inducers for 21 days. Following removal of the culture medium, the cells were fixed with a 4% paraformaldehyde solution for 30 min and washed three times with ultrapure water. Then, Alizarin Red staining (ARS) solution was added for suitable staining, the excess dye was removed. Finally, the stained cells were washed twice with ultrapure water and observed under an inverted microscope.

### Reverse transcription-quantitative polymerase chain reaction

BMSC and MC3T3-E1 cells were seeded in 6-well plates at a density of 2 × 10^4^ per well, respectively, consistent with the treatments of the above experiments. Osteogenic differentiation-related gene expression was analyzed at Day 7 and Day 14. After collecting the cells, TRIzol (Bio-rad, USA) was applied for cell lysis and total RNA extraction. Subsequently, the extracted RNA was reverse transcribed using the iScript cDNA Synthesis Kit (Bio-rad, USA). SsoFast EvaGreen Supermixes (Bio-rad, USA) were then used to detect the levels of osteogenic gene expression in the cells. The specific primers for the relevant genes are listed in Table 1.

**Table 1.** The primers sequences for real-time PCR

Gene subtype	Oligonucleotide primers (5′–3′)
GAPDH (Norway rat)	AGACAGCCGCATCTTCTTGT CCGATACGGCCAAATCCGTT
BMP-2 (Norway rat)	CCCCCTATATGCTCGACCTG TCCTCGATGGCTTCTTCGTG
RUNX-2 (Norway rat)	GACCAACCGAGTCATTTAAGGC TTTGACGCCATAGTCCCTCC
OCN (Norway rat)	CCTCAACAATGGACTTGGAGCC GAAGCCAATGTGGTCCGCTA
GAPDH (*Mus musculus*)	GCCTCCTCCAATTCAACCCTT TGTCTACGGGACGAGGAAAC
BMP-2 (*M. musculus*)	TTCCATCACGAAGAAGCCGT GAAACTCGTCACTGGGGACA
RUNX-2 (*M. musculus*)	GGGAACCAAGAAGGCACAGA GGATGAGGAATGCGCCCTAA
OCN (*M. musculus*)	GCTCACTCTGCTGACCCTG GGGACTGAGGCTCCAAGGTA

### Statistical analyses

Data in this paper were expressed as mean ± SD deviation of the three groups ($\bar{x}$ ± s), and one-way analysis of variance was used for comparison between groups. The significance level was set as *P* < 0.05 (*), *P* < 0.01 (**), *P* < 0.001 (***).

## Results

### Characterizations of MCAPS

**Figure 1.**
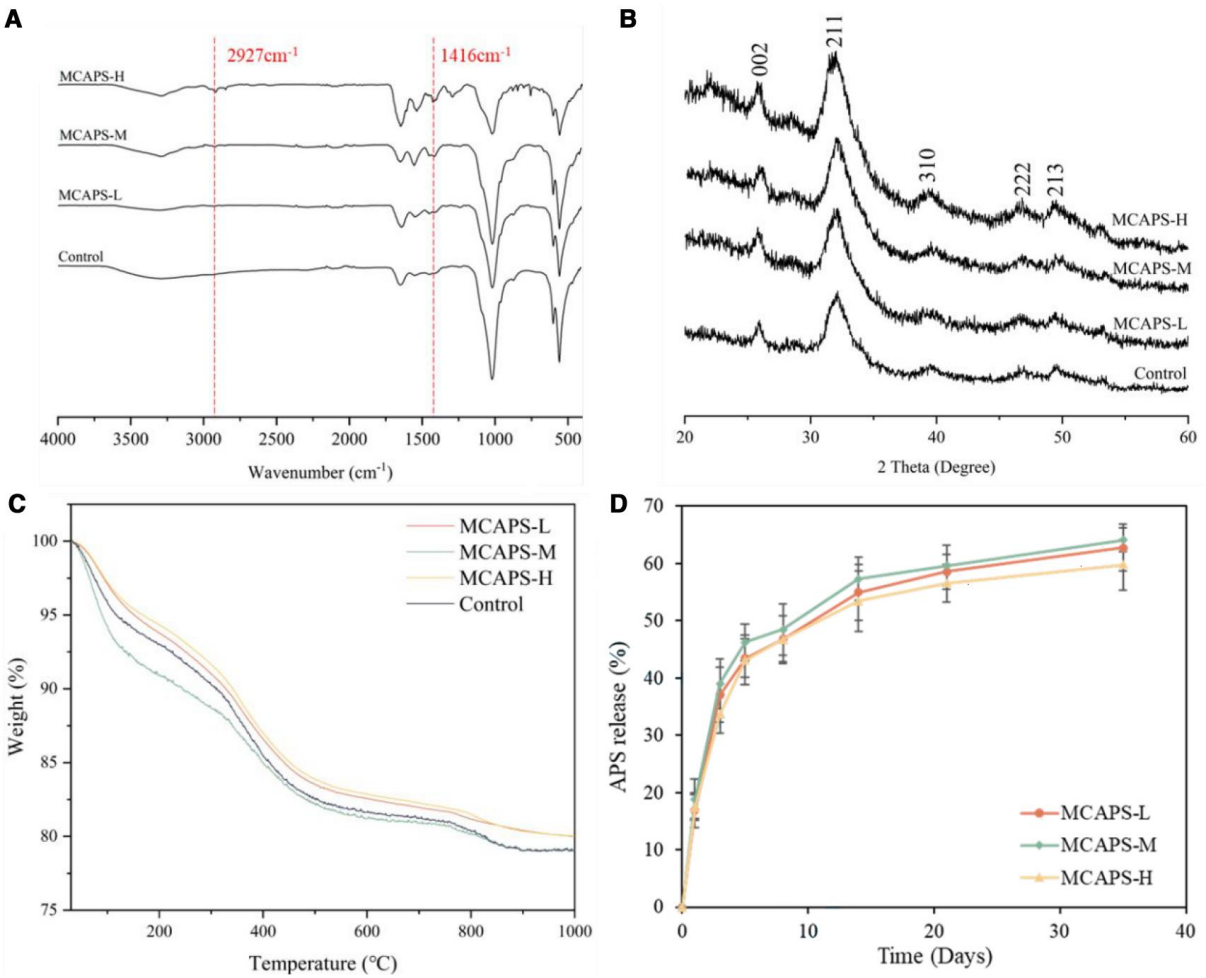
(**A**) The FTIR spectrum of MCAPS and Control. (**B**) XRD. (**C**) TGA diagram of MCAPS. (**D**) APS release spectrum of MCAPS.

The IR spectra (Figure 1A) show that the peaks at 1647, and 1540 cm^−1^ were derived from the characteristic vibrations of the amide bonds of the collagen macromolecules in MCAPS fibrils. The absorption bands at 556, 602 and 1015 cm^−1^ correspond to the typical signals of the ${\text{PO}}_{4}^{3-}$ group in apatite, confirming the presence of calcium phosphate minerals. The bands at 3378, 2927, 1626, 1417 and 1050 cm^−1^ attributed to APS were legibly identified [40, 42], some of whose peaks overlapping with those of mineralized collagen macromolecules in MCAPS. The absorption peaks at 2927 and 1416 cm^−1^ are characteristic vibrations of polysaccharides, demonstrating the presence of APSs in MCAPS.

The XRD patterns (Figure 1B) produced from the composite fibrils of MCAPS reveal the characteristic diffraction peaks of (002), (211), (310), (222) and (213), which were derived from hydroxyapatite crystals, confirming the existence of hydroxyapatite mineral phase in MCAPS [43, 44]. Furthermore, the XRD spectrogram exhibits that there is no significant difference between the MCAPS group and the Control group in terms of their characteristic diffraction peaks, indicating no substantial effect of the introduction of APSs on the formation of the hydroxyapatite crystal phase in MCAPS.

TGA was applied to analyze the weight loss of MCAPS composites under elevated temperatures for exploring the ratio of organic to inorganic components in the composite fibrils (Figure 1C). After undergoing a temperature rise from 30°C to 1000°C, the residual masses of the MCAPS-L, MCAPS-M, MCAPS-H and Control groups were 80.02%, 79.07%, 80.03% and 79.07%, respectively. There was little difference in the mineral phase content between the MCAPS and the Control groups. The proportions of organic part and water in each group were around 20% while the mineral portion were around 80%.

As shown by the *in vitro* release curves (Figure 1D), the release of the APS from the materials of MCAPS was at a faster rate during the first 3 days, and the cumulative release of MCAPS-L, MCAPS-M and MCAPS-H at Day 3 reached 37.08 ± 4.33%, 39.02 ± 4.74% and 33.72 ± 3.37%, respectively. In the following time, the release rate gradually decreased, and at Day 35, the cumulative release of MCAPS-L, MCAPS-M and MCAPS-H reached 62.76 ± 2.04%, 64.12 ± 4.03% and 59.75 ± 4.32%, respectively. The release results suggest that the materials of MCAPS could provide a sustained release of APSs over a long period of time. The content of APS in MCAPS-L, M and H groups were 145.720 ± 0.135 μg/g, 293.675 ± 0.117 μg/g, 589.937 ± 0.203 μg/g, respectively.

### Microscopic topography and energy spectrum analyses of MCAPS

**Figure 2.**
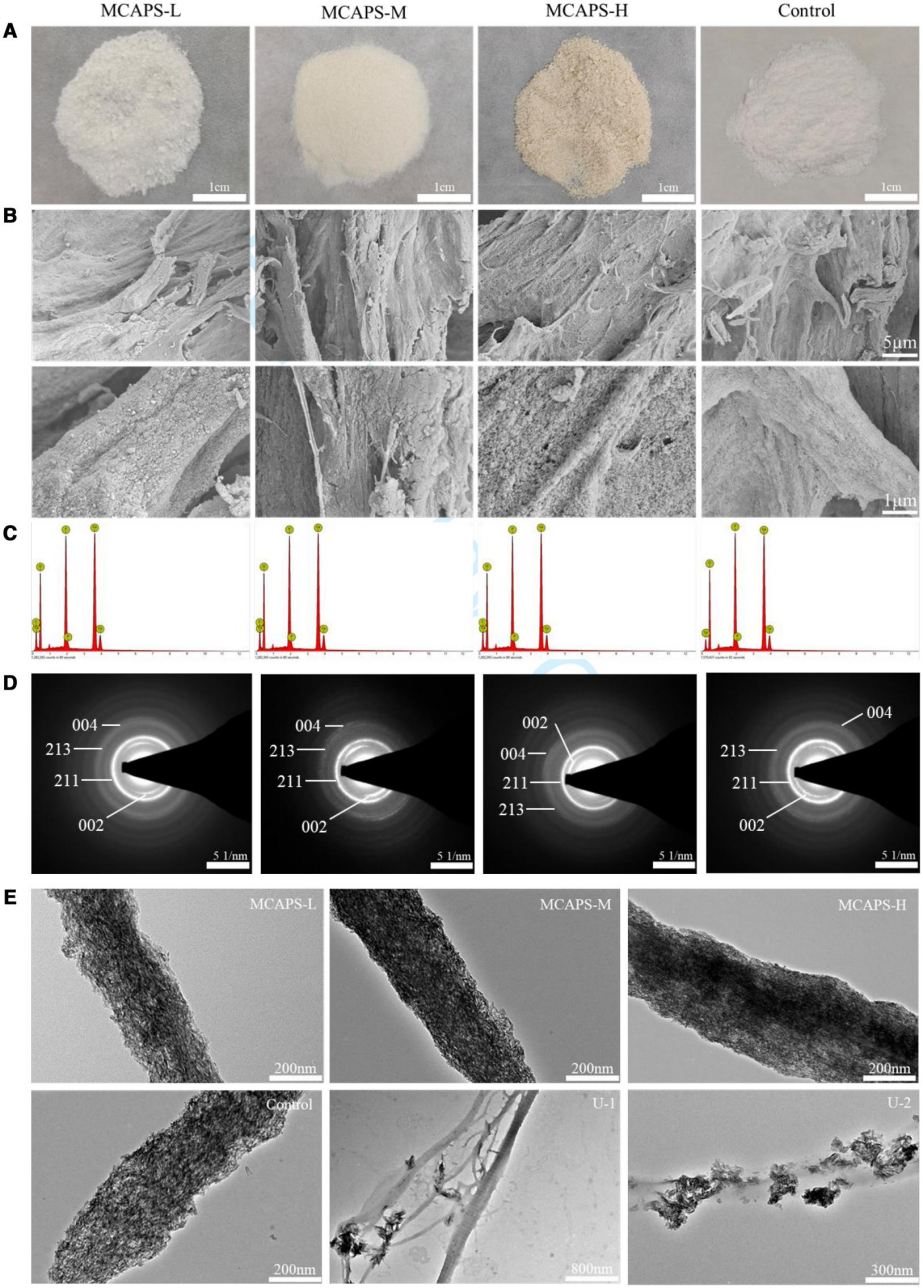
Powder appearance (**A**), SEM images (**B**), EDS analyses (**C**), SAED (**D**) and TEM images (**E**) of the biomineralized collagen fibrils of MCAPS and Control groups, unsuccessfully biomineralized collagen fibrils (U-1and U-2).

The SEM images in Figure 2B present the structural relationship between the collagen fibrils and the apatite minerals in both MCAPS and Control groups, that is all of the collagen fibrils were thoroughly mineralized by a layer of relatively uniform apatite nanocrystals, which is very similar to the biomimetic mineralization produced by polymer-induced liquid precursor (PILP) mineralization process. Meanwhile, there are no obvious differences between the MCAPS and the Control group in term of the mineralization microstructures, indicating that the existence of APS had no substance effect on the biomineralization state for collagen fibrils.

The TEM images (Figure 2E) of all MCAPS groups and Control group clearly exhibit the full and relatively uniform binding of hydroxyapatite nanocrystals with the collagen fibrils in the materials of all MCAPS and Control groups, with the orientation of the calcium phosphate crystals parallel to the long axis of the collagen fibrils, which is similar to the secondary level of bone tissue microstructures. In contrast to this biomineralization, there is only a small quantity of apatite nanoparticles randomly scattering on the unsuccessfully biomineralized collagen fibrils (Figure 2E U-1 and U-2) far from forming a complete and relatively uniform layer of mineral nanocrystals. The EDS analyses revealed the Ca/P ratios for MCAPS-L, MCAPS-M, MCAPS-H and Control were 1.533 ± 0.034, 1.540 ± 0.056, 1.539 ± 0.038 and 1.537 ± 0.035, respectively (Figure 2C), which are very close to that of native bone tissue. The SAED results (Figure 2D) demonstrate the diffraction loops of HA crystals on mineralized collagen fibers at (002), (211), (213) and (004) crystal planes for each group of MCAPS. This further corroborates the presence of HA crystals in MCAPS materials. Furthermore, the diffraction loops at (002) and (004) exhibited a discernible orientation, indicating that the long axis of the HA crystals was aligned with the direction of the long axis of the mineralized collagenous protofibrils. This is consistent with the arrangement and orientation of hydroxyapatite in natural bone tissue, thereby further verifying that MCAPS achieves bionic mineralization of secondary microstructures in natural bone tissue.

### Cytocompatibility of MCAPS materials

**Figure 3.**
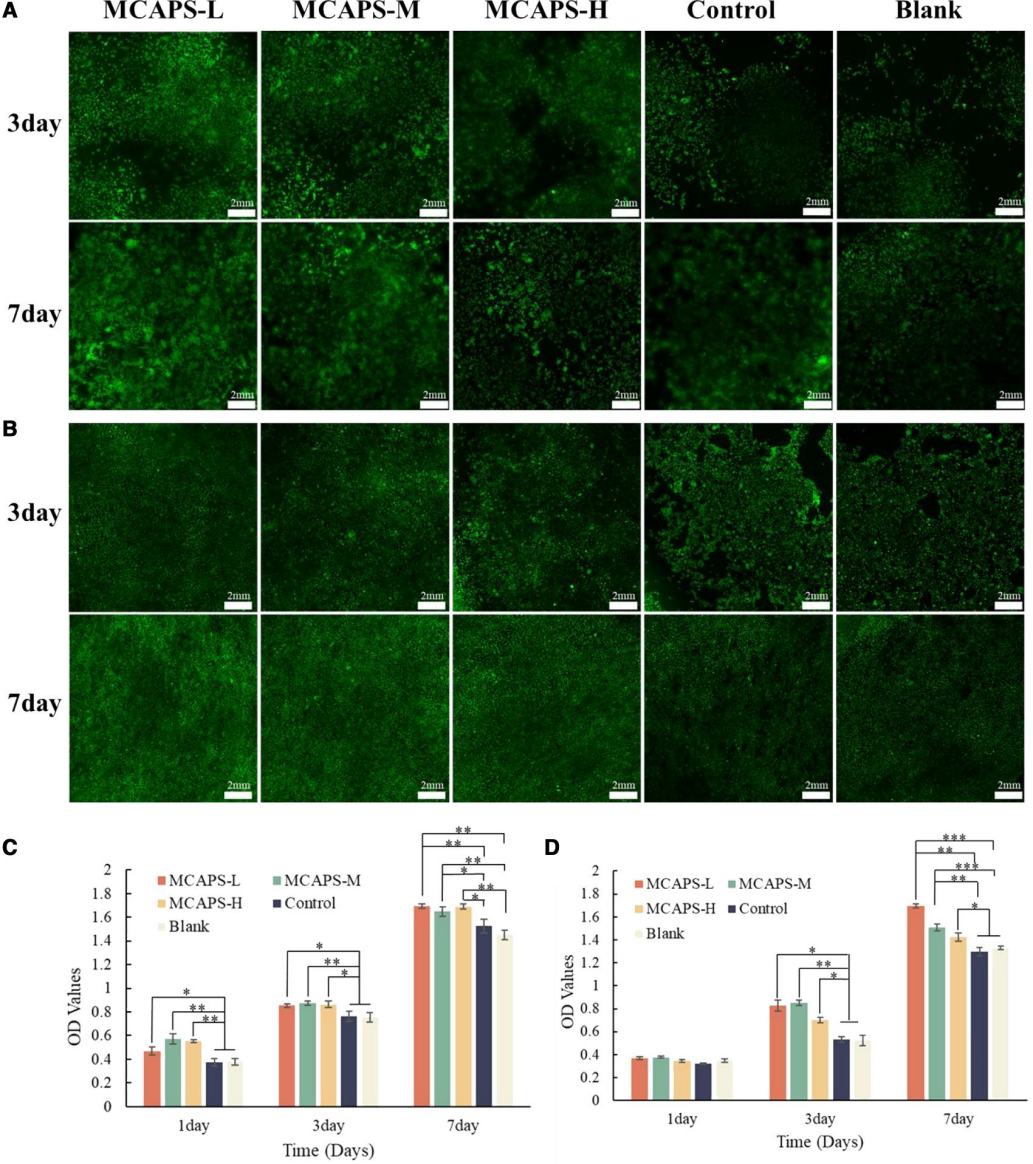
The live/dead staining of BMSC (**A**) and MC3T3-E1 (**B**). Cell viability of BMSC (**C**) and MC3T3-E1 (**D**).

The cells were subjected to CCK-8 assay at Days 1, 3 and 7, as well as live/dead staining at Days 3 and 7 to determine the cell proliferation. For BMSC (Figure 3C), at Day 1, the absorbance for MCAPS-L, MCAPS-M, MCAPS-H, Control and Blank groups was 0.469 ± 0.035, 0.572 ± 0.043, 0.553 ± 0.013, 0.376 ± 0.03 and 0.378 ± 0.025, respectively. The cell activity of MCAPS groups was higher than that of the Control and Blank groups (*P* < 0.05). At Day 3, the absorbance for MCAPS-L, MCAPS-M, MCAPS-H, Control and Blank groups was 0.853 ± 0.016, 0.875 ± 0.02, 0.865 ± 0.026, 0.764 ± 0.044 and 0.755 ± 0.04, respectively, with higher cell activity of MCAPS groups than that of the Control and Blank groups (*P* < 0.05). At Day 7, the absorbance for MCAPS-L, MCAPS-M, MCAPS-H, Control and Blank groups was 1.697 ± 0.019, 1.649 ± 0.038, 1.689 ± 0.061, 1.525 ± 0.057 and 1.451 ± 0.038, respectively, also with higher cell activity of MCAPS groups than that of the Control and Blank groups (*P* < 0.05). For the MC3T3-E1 cells (Figure 3D), although there was no significant difference of absorbance detected among the groups after 1 day of incubation. However, until Day 3, the absorbance values for the MCAPS-L, MCAPS-M and MCAPS-H groups were 0.827 ± 0.046, 0.851 ± 0.027 and 0.699 ± 0.023, respectively, while the values for the Control and Blank groups were 0.533 ± 0.025 and 0.523 ± 0.04, respectively, indicating that the cell viability of the MCAPS groups was significantly higher than that of the Control and Blank groups (*P* < 0.05). Particularly, the MCAPS-L and MCAPS-M groups showed a more pronounced promoting effect on cell proliferation. At Day 7, the absorbance values for the MCAPS-L, MCAPS-M, MCAPS-H, Control and Blank groups were 1.569 ± 0.035, 1.509 ± 0.032, 1.425 ± 0.037, 1.296 ± 0.035 and 1.329 ± 0.015, respectively. The cell viability of both MCAPS-L and MCAPS-M groups was higher than that of the Control and Blank groups (*P* < 0.01) due to the promoting effect on cell proliferation was more favorable in the MCAPS-L and MCAPS-M groups.

Figure 3A and B show that both BMSC and MC3T3-E1 had a quite higher viable cell ratio (green fluorescence) than the Control and Blank groups after 3 and 7 days of culture. The CCK-8 results for BMSC and MC3T3-E1 at Day 1, 3 and 7 (Figure 3C and D) suggest that the APS component in the composite fibrils of MCAPS materials exhibited promoting cell proliferation. The live/dead staining results for BMSC and MC3T3-E1 (Figure 3A and B) at Day 3 and 7 demonstrate that the cell viability of the MCAPS groups is higher than that of the Blank and Control groups, further indicating the beneficial efficacy of the APS component in MCAPS materials on the proliferation of BMSC and MC3T3-E1.

### Osteogenesis promotion by MCAPS *in vitro*

**Figure 4.**
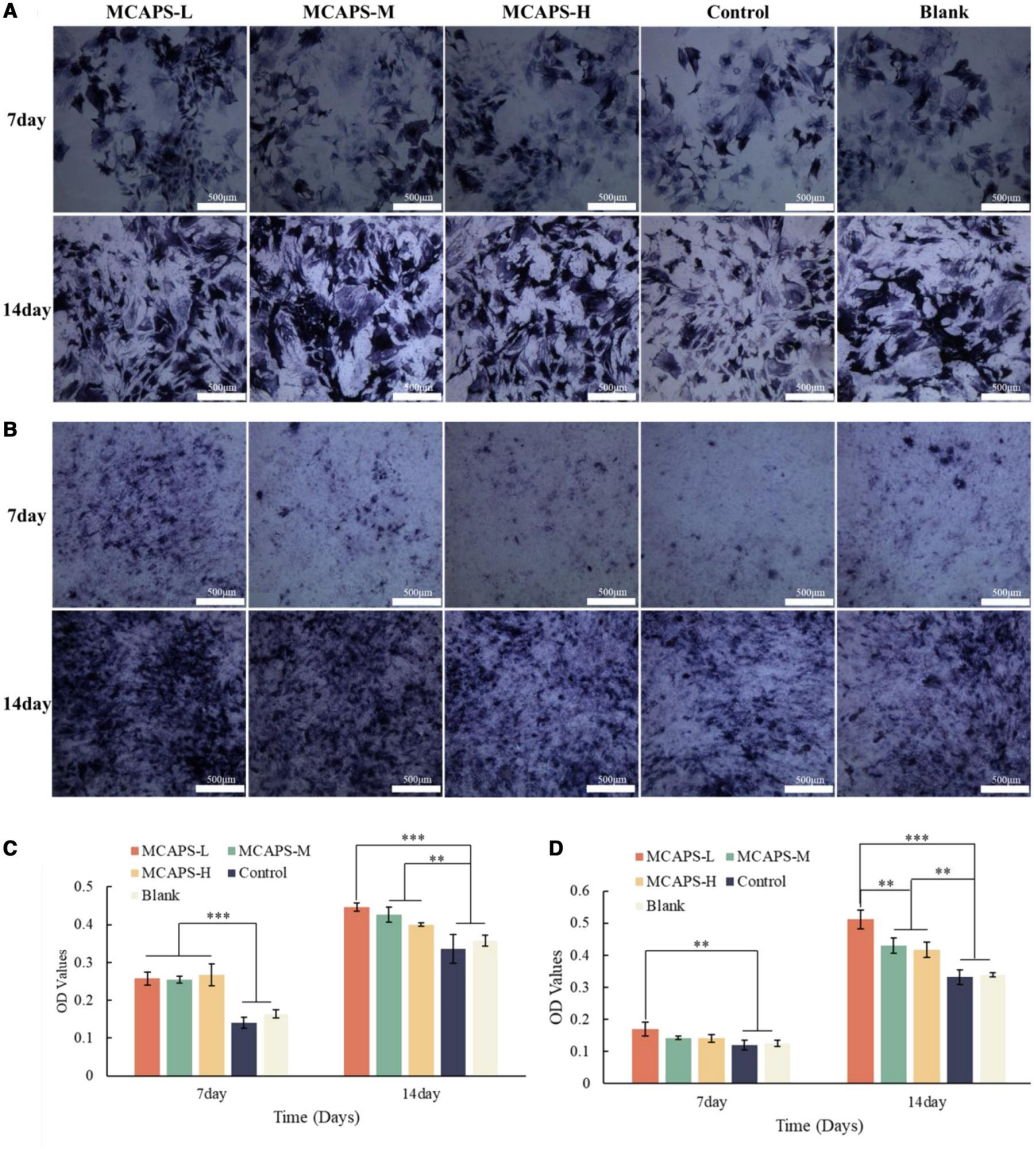
The ALP staining images of BMSC (**A**) and MC3T3-E1 (**B**) at Days 7 and 14. The ALP activity detection of BMSC (**C**) and MC3T3-E1 (**D**) at Days 7 and 14.

Alkaline phosphatase (ALP) is closely related to osteogenic differentiation and is one of the main characteristics of osteoblast differentiation. After 7 days of BMSC culture (Figure 4C), the ALP was quantitatively detected for the group of MCAPS-L, MCAPS-M, MCAPS-H, Control and the Blank, obtaining the absorbance values of 0.258 ± 0.017, 0.254 ± 0.008, 0.267 ± 0.029, 0.14 ± 0.015 and 0.164 ± 0.01, respectively. The ALP content of each MCAPS group was higher than that of the Control and Blank groups (*P* < 0.01). After 14 days of culture (Figure 4C), the absorbance of the ALP quantitatively acquired from the group of MCAPS-L, MCAPS-M, MCAPS-H, Control and the Blank was 0.436 ± 0.023, 0.429 ± 0.024, 0.43 ± 0.012, 0.336 ± 0.037 and 0.358 ± 0.014, respectively. These results demonstrate that the composites of MCAPS could enhance the ALP activity of BMSC, consequently promoting the osteogenic differentiation of BMSC.

For MC3T3-E1 (Figure 4D), after 7 days of incubation, the ALP quantification by absorbance of the group of MCAPS-L, MCAPS-M, MCAPS-H, Control and the Blank was 0.170 ± 0.021, 0.143 ± 0.006, 0.141 ± 0.012, 0.120 ± 0.015 and 0.126 ± 0.01, respectively. Only the ALP content of MCAPS-L group was significantly higher than that of the Control and Blank groups. At Day 14 of culture, the absorbance of the ALP corresponding to MCAPS-L, MCAPS-M, MCAPS-H, Control and the Blank groups was 0.512 ± 0.029, 0.426 ± 0.024, 0.416 ± 0.024, 0.332 ± 0.023 and 0.339 ± 0.007, respectively. The ALP content of all MCAPS groups was higher than that of the Control and Blank groups, with MCAPS-L showing the most significant promotion of ALP. These results demonstrate that the materials of MCAPS have the capability of enhancing the ALP activity of MC3T-E1 and promoting osteogenic differentiation.

The results of ALP staining showed that at Days 7 and 14 of culture, the staining intensity of the MCAPS group of BMSC cells (Figure 4A) was higher than that of the Control and the Blank group, further confirming the promoting efficiency of MCAPS on BMSC osteogenic differentiation. Similarly, the staining intensity of MCAPS for MC3T3-E1 (Figure 4B) at Days 7 and 14 was also higher than that of the Control and the Blank group, further confirming the promoting effect of MCAPS on MC3T3-E1 osteogenic differentiation.

**Figure 5.**
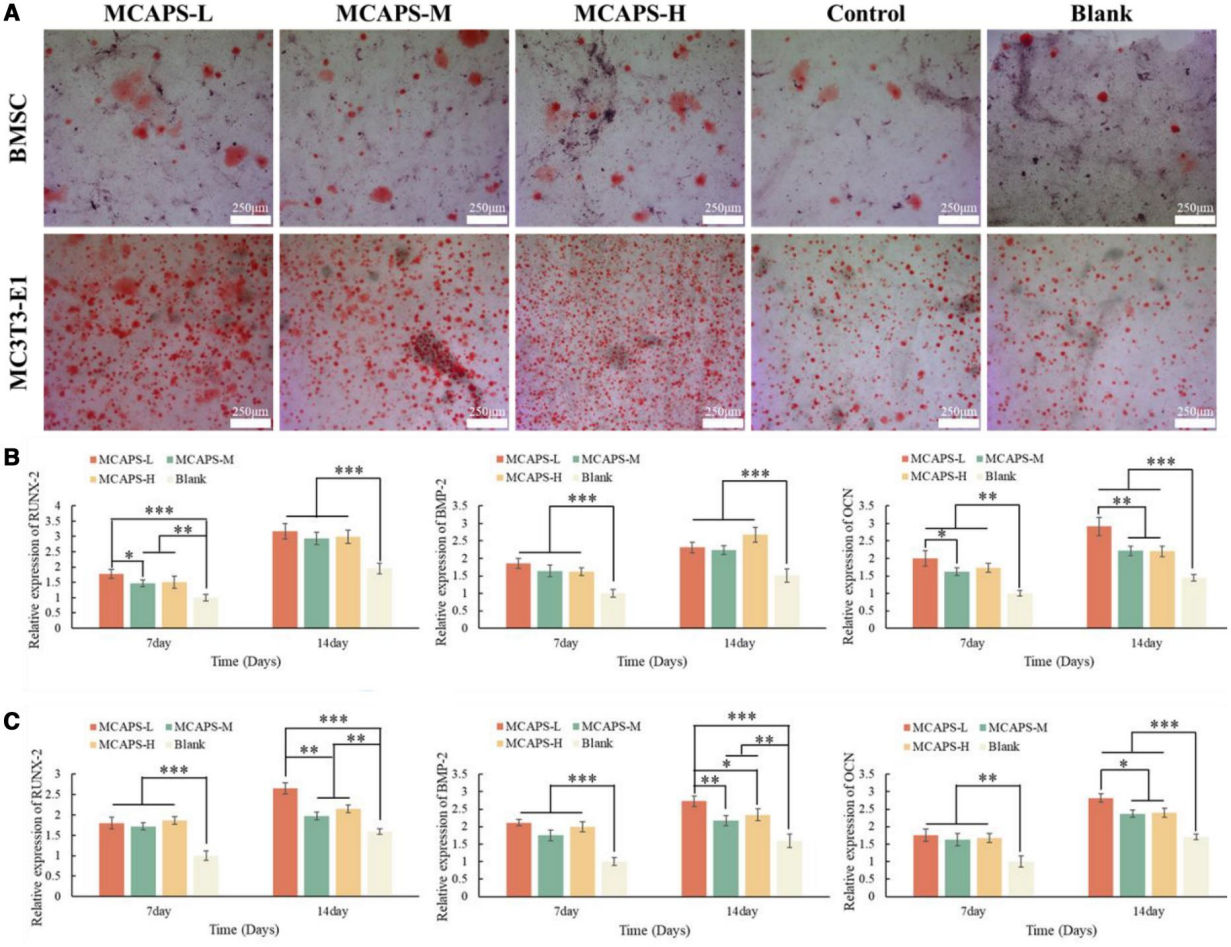
(**A**) The images of ARS of BMSC and MC3T3-E1 at Day 21. The relative mRNA expression of Runx-2, BMP-2 and OCN in BMSC (**B**) and MC3T3-E1 (**C**) at Days 7 and 14.

When the cells are in the late stage of osteogenic differentiation, mineralization occurs and calcium nodules (CN) are generated, and Alizarin Red can be easy to stain the calcium nodules to evaluate the osteogenic differentiation-promoting properties of the material. The results of ARS (Figure 5A) showed that the number of calcium nodules of BMSC cells in each group of MCAPS was higher than that of the Control and Blank groups, indicating that the materials of MCAPS have excellent bone-enhancing properties. Meanwhile, for MC3T3-E1, observation under the microscope revealed that the red staining of MCAPS groups was darker than that of the Control and Blank groups, which further identified the excellent bone-conducive effects of the materials of MCAPS.

Runx-2, as a crucial regulator in the formation of osteoblasts and chondrocytes, is involved in the regulation of multiple steps of bone development, and interacts with various osteogenesis-related factors in the osteogenic differentiation signaling pathway to regulate the osteogenic differentiation of osteoblasts. This regulation is essential for the development and maturation of both osteoblasts and chondrocytes. At Day 7, the relative expression levels of Runx-2 in BMSC cells for MCAPS-L, MCAPS-M, MCAPS-H and the Blank group (Figure 5B) were 1.769 ± 0.143, 1.474 ± 0.111, 1.505 ± 0.199 and 1.000 ± 0.109, respectively. At Day 14, the relative expression levels of Runx-2 in BMSC cells for MCAPS-L, MCAPS-M, MCAPS-H and the Blank group were 3.175 ± 0.249, 2.935 ± 0.192, 2.989 ± 0.221 and 1.949 ± 0.178, respectively. At both time points, the relative expression levels of Runx-2 in the MCAPS groups were significantly higher than those in the Blank group (*P* < 0.01). At Day 7, the expression level of MCAPS-L was higher than that of MCAPS-M (*P* < 0.05), while at Day 14, there was no significant difference in expression levels among the MCAPS groups. At Day 7, the relative expression levels of Runx-2 in MC3T3-E1 cells (Figure 5C) were 1.803 ± 0.141, 1.721 ± 0.085, 1.862 ± 0.096 and 1.000 ± 0.114 for MCAPS-L, MCAPS-M, MCAPS-H and the Blank group, respectively. At Day 14, the relative expression levels were 2.651 ± 0.131, 1.972 ± 0.089, 2.149 ± 0.094 and 1.591 ± 0.069 for the same corresponding groups. At both time points of Days 7 and 14, the relative expression levels of Runx-2 in the MCAPS groups were significantly higher than those in the Blank group (*P* < 0.01). There were no significant differences in term of expression levels among the MCAPS groups at Day 7, but at Day 14, the expression level of MCAPS-L group was higher than that of MCAPS-M and MCAPS-H group (*P* < 0.01).

BMP-2 also plays a critical role in bone tissue development by promoting the differentiation of osteoblasts and the synthesis and secretion of extracellular matrix. It also acts as an initiating regulatory factor to promote the formation of new bone. The relative expression levels of BMP-2 in BMSC cells at Day 7 for MCAPS-L, MCAPS-M, MCAPS-H, and the Blank group (Figure 5B) were 1.857 ± 0.146, 1.643 ± 0.166, 1.621 ± 0.107 and 1.000 ± 0.108, respectively. At Day 14, the relative expression levels of BMP-2 for MCAPS-L, MCAPS-M, MCAPS-H and the Blank group were 2.313 ± 0.149, 2.235 ± 0.127, 2.676 ± 0.214, 1.505 ± 0.192. At both Days 7 and 14, the relative expression levels of BMP-2 from the MCAPS groups were significantly higher than that from the Blank group (*P* < 0.001), and there were no significant differences among the MCAPS groups. At Day 7, the relative expression levels of BMP-2 in MC3T3-E1 cells for MCAPS-L, MCAPS-M, MCAPS-H and the Blank group (Figure 5C) were 2.114 ± 0.083, 1.753 ± 0.154, 1.996 ± 0.142 and 1.000 ± 0.113, respectively. At Day 14, the relative expression levels of BMP-2 from MCAPS-L, MCAPS-M, MCAPS-H and the Blank group (Figure 5C) were 2.732 ± 0.153, 2.179 ± 0.144, 2.341 ± 0.169 and 1.598 ± 0.193, respectively. At these two time points, the relative expression levels of the BMP-2 from all MCAPS groups were significantly higher than that from the Blank group (*P* < 0.01). No significant differences among the MCAPS groups at Day 7, while at Day 14, the relative expression level of the BMP-2 in MCAPS-L group was higher than that in MCAPS-M (*P* < 0.01) and MCAPS-H groups (*P* < 0.05).

For bone tissue formation, osteocalcin (OCN) is an important regulatory factor, participating in the biomineralization process inside organism. The relative expression levels of the OCN in BMSC cells at Day 7 for MCAPS-L, MCAPS-M, MCAPS-H and the Blank group (Figure 5B) were 2.005 ± 0.223, 1.621 ± 0.112, 1.729 ± 0.129 and 1.000 ± 0.075, respectively, and were 2.915 ± 0.259, 2.219 ± 0.132, 2.199 ± 0.146 and 1.447 ± 0.093, respectively at Day 14. At least within the culture period of 14 days, the relative expression levels of OCN in the MCAPS groups were significantly higher than those in the Blank group (*P* < 0.01). At Day 7, the expression level detected from MCAPS-L group was higher than that from MCAPS-M group (*P* < 0.05), and at Day 14, the expression level from MCAPS-L group was higher than that from MCAPS-M and MCAPS-H group (*P* < 0.01). The MC3T3-E1 cells expressed relative levels of OCN (Figure 5C) in MCAPS-L, MCAPS-M, MCAPS-H and the Blank group at Day 7 were 1.757 ± 0.178, 1.628 ± 0.180, 1.678 ± 0.126 and 1.000 ± 0.161, respectively, while at Day 14, the relative expression levels of the OCN in MCAPS-L, MCAPS-M, MCAPS-H and the Blank group were 2.815 ± 0.119, 2.367 ± 0.107, 2.395 ± 0.131 and 1.709 ± 0.084, respectively. During the experimental period of 14 days, the OCN relative expression levels of all MCAPS groups were significantly higher than those of the Blank group (*P* < 0.01). No significant differences among the MCAPS groups were detected at Day 7, whereas at Day 14, the expression level of the OCN was monitored to be higher in MCAPS-L group than that in MCAPS-M and MCAPS-H group (*P* < 0.05).

The osteogenic properties of the composite materials of MCAPS were explored via the quantitation and staining of ALP, ARS and reverse transcription-quantitative polymerase chain reaction (RT-qPCR). These results demonstrate that the composites of MCAPS have the efficacy of enhancing the ALP activity in BMSC and MC3T3-E1 cells, promoting the osteogenic differentiation and the calcium deposition of BMSC and MC3T3-E1. The results of the RT-qPCR had given the evidence that the composites of MCAPS can promote the upregulation expression of the osteogenic-related gene including Runx-2, BMP-2 and OCN, further validating the promotion effects of MCAPS on the osteogenic differentiation of BMSC and MC3T3-E1, thus facilitating bone tissue regeneration. All the results of RT-qPCR showed no statistical differences in the expression levels of osteogenic-related genes between the Blank and Control groups (Figure S1).

## Discussion

Biomineralized collagen composite material is preferable for bone-repair due to its biomimetic microstructures and components similar to native bone tissue, as well as the excellent biocompatibility and biodegradability. The biomimetic mineralization *in vitro* of intrafibrillar mineralization, which is the closest to the secondary level of bone microstructures, was first achieved by the approach of PILP [45, 46]. In this study, we synthesized a material of MCAPS, which are composite collagen fibrils mineralized by hydroxyapatite binding APS, with the characteristic microstructure of intrafibrillar mineralization by utilizing the method of co-precipitation. TEM characterizations (Figure 2E) give the details about the structural relationship between the collagen fibrils and the mineral nanocrystals, that is all of the mineral nanocrystals had grown within the collagen fibril with their orientation parallel to the long axis of the fibrils. This binding state demonstrates that the growth of the mineral crystals entirely occurred in the collagen fibrils as organic templates, consequently achieving the intrafibrillar mineralization of the collagen fibrils, and on this basis, a relatively uniform and continuous mineralization layer was further developed for the collagen fibrils, as exhibited by the SEM (Figure 2B). In the method of PILP process, a liquid amorphous mineral precursor fluid can be formed by the binding of calcium ions to the abundant carboxyl groups on the macromolecular chains. This precursor fluid can infiltrate into the interstices and grooves of preformed collagen fibrils by capillary action, which upon crystallization after dehydration, achieving intrafibrillar mineralization. While in the *in-situ* co-precipitation method, the synergy between the self-assembly of collagen macromolecules and the nucleation and growth of nanosized hydroxyapatite crystals was achieved by optimizing and controlling the reaction parameters such as supply rate of calcium and phosphate ions, temperature and stirring rate etc. That is, while the collagen molecules were self-assembling, the calcium phosphate crystal nuclei were synchronously generated, aggregated and grown on the collagen microfibrils to form nanocrystals, so as to realize a relatively uniform and continuous mineralization layer of apatite nanocrystals inside and outside the collagen fibrils. PILP synthesis method has a longer synthesis period. Additionally, it is prone to issues such as uneven and discontinuous mineralization due to local variations in the penetration of the mineral precursor liquid, making PILP method unsuitable for large-scale production of such MCF. On the other hand, the *in-situ* co-precipitation method allows for the simultaneous nucleation, aggregation and growth of calcium phosphate crystallite on the oligomer of self-assembling collagen molecules during the synthesis process, resulting in the formation of relatively uniform and continuous mineral layer within and onto the collagen fibrils. Therefore, this method facilitates the large-scale production of biomineralized collagen fibrils in a short period. In contrast to biomimetic intrafibrillar mineralization, only a small quantity of apatite nanocrystals randomly scattered on the collagen fibrils, implying a failure of intrafibrillar biomimetic mineralization. And the SAED results further demonstrate the presence of HA crystals in MCAPS and reveal that the orientation of HA crystals is similar to that observed in the secondary microstructure of natural bone. The mineral phase of the materials of MCAPS were identified as typical weak-crystalline hydroxyapatite with a Ca/P ratio of about 1.53 (Figures 1A and B and 2C), very close to that of native bone tissue [21]. APSs have better water solubility and, according to existing literatures, possess osteogenesis-promoting effects [39, 47]. Therefore, we designed a strategy that APSs are present throughout the entire synthesis process of the biomineralized collagen fibrils. This route allows for the spontaneous incorporation of APSs into the biomimetic MCF, resulting in a material with the desired osteogenic properties. The coexistence of collagen and APS embedded in apatite layer was also confirmed by their respective characteristic peaks in IR spectra. In the materials of MCAPS, the inorganic mineral part accounts for around 80%, which can be adjusted by changing the feeding ratio.

For the low, medium and high content of APS in the material of MCAPS, the existence of APS had no any substantial effects on the intrafibrillar mineralization degree, micromorphology and even Ca/P ratio of the composite collagen fibrils of MCAPS, which is most likely attributed to the too small molecular weight of APS. The bound APS in the composite collagen fibrils of the MCAPS exhibited a sustained release *in vitro* over a period of more than 1 month, although a rapid release occurred in the first 3 days. And different loading amount of APS had no distinct impact on the release rate of the APS from MCAPS.

All results of the experiments investigating the effects of the MCAPS on osteoblast demonstrated the bone regeneration-promoting efficacy of the MCAPS, which was mainly enhanced by the APS loaded in the MCAPS. This bone regeneration-promoting efficacy of the MCAPS was manifested on three levels.

On the level of cell behaviors, the proliferation of BMSC and MC3T3-E1 cells was promoted by the materials of MCAPS, with a strongest promotion effect from the MCAPS-L group. BMSCs and MC3T3-E1 are widely used for investigating the osteogenic potential of materials. BMSCs are multipotent stem cells with ability to differentiate into various cell types, including osteoblasts, adipocytes and chondrocytes. In the process of osteogenesis, BMSCs not only can differentiate into osteoblasts to promote the repair of bone tissue but also can inhibit inflammatory responses and modulate immunity to a certain extent, which is beneficial for the healing of bone defects [48]. MC3T3-E1, a mouse embryonic osteoblast precursor cell line, has the ability to differentiate into osteoblasts and osteocytes. They can produce collagen and perform extracellular calcium deposition during osteogenesis, exhibiting various characteristics of osteoblasts. This cell line is widely used in the study of osteoblast differentiation and the mechanisms of bone formation. Therefore, we had chosen BMSCs to explore the ability of MCAPS materials to promote the osteogenic differentiation of BMSCs and simultaneously to promote the osteogenic differentiation process of MC3T3-E1. At the level of the osteogenic properties, MCAPS showed the capability of significantly enhancing the ALP activity and the number of calcium nodules in BMSC and MC3T3-E1. In the process of osteoblast differentiation, the ALP activity within osteoblasts is a marker for the early stage of osteogenesis. With the further osteogenic differentiation of osteoblasts, calcium ions are deposited to form extracellular calcium nodules, which are the symbol of the differentiation and maturity of osteoblasts, and also the main morphological characteristics of the osteoblast in performing osteogenic function. Accordingly, basing on the activity level of ALP and the number of calcium nodules, the capability of MCAPS material promoting the osteogenic differentiation of BMSC and MC3T3-E1can be identified. The experimental result is: MCAPS can upregulate the expression level of both ALP and calcium nodules in BMSC and MC3T3-E1, which respectively serve as the markers for the early and late stage of osteogenesis. To further confirm the osteogenic performance of MCAPS from the molecular biology level, RT-qPCR was applied to detect the expression levels of three osteogenic related genes, RUNX-2, BMP-2 and OCN. RUNX-2 is responsible for regulating the differentiation and maturation of osteogenesis-relevant cells. BMP-2 plays an important role in promoting the differentiation and maturation osteogenesis-relevant cells, participating in the growth and reconstruction of bone and cartilage. OCN is a specific protein of bone tissue and plays an important role in regulating bone tissue mineralization. The expression levels of RUNX-2, BMP-2 and OCN genes in all groups of MCAPS were significantly higher than the Blank group. Over a long period of 14 days of culture, MCAPS-L showed better effects in upregulating ALP activity, calcium nodule quantity and promoting the expression of osteogenic-related genes when compared to MCAPS-M and MCAPS-H. This is because APS can promote the proliferation and differentiation of osteoblasts at low concentrations, while high concentrations of APS are not conducive to the proliferation and differentiation of osteoblasts, and may even inhibit these processes [47, 49].

## Conclusion

From a biomimetic inspiration, a functional biomineralized collagen fibrillar material with a structure similar to the secondary level of bone tissue microstructure was successfully synthesized *in vitro* for exploring an innovative bone regeneration-repair material. A relatively uniform and intrafibrillar mineralization of the collagen fibrils was achieved by simultaneous occurrence of the self-assembly of collagen macromolecules with the growth of the mineral nuclei on the surface of the collagen microfibrils, mimicking the intrafibrillar mineralization of native bone, which was confirmed by TEM and SEM. Additionally, water soluble APS was introduced to participate in the complete growth process of these biomineralized collagen fibrils, obtaining a novel kind of mineralized collagen-APS composite fibril materials (MCAPS). The existence of the bound APS within the MCAPS fibrils was identified by IR. The APS of each MCAPS group can be in sustained release *in vitro* over a period of more than 1 month. The mineral phase of the materials of MCAPS was typical weak-crystalline hydroxyapatite with a Ca/P ratio of about 1.53, very close to that of native bone tissue.

The bone regeneration-promoting efficacy of the MCAPS was demonstrated from three levels. For cell behaviors, the proliferation of BMSC and MC3T3-E1 cells was promoted by the materials of MCAPS. As for the osteogenic properties, MCAPS showed a significant enhancement for the ALP activity and the number of calcium nodules in BMSC and MC3T3-E1. Three osteogenic related genes of RUNX-2, BMP-2 and OCN were detected via RT-qPCR to further confirm the osteogenic performance promotion of MCAPS from the molecular biology level. A long period of 14 days of culture experiment showed that MCAPS-L exerted a more excellent efficacy in upregulating ALP activity and calcium nodule quantity together in promoting the expression of osteogenic-related genes over MCAPS-M and MCAPS-H.

## Supplementary Material

rbae070_Supplementary_Data
