# Supplementary material for: In situ co-deposition synthesis for collagen-Astragalus polysaccharide composite with intrafibrillar mineralization as potential biomimetic-bone repair materials
Source: Regen Biomater. 2024 Jun 21;11:rbae070. doi: 10.1093/rb/rbae070 (PMC11254354; doi:10.1093/rb/rbae070)
Supplement: rbae070_Supplementary_Data [file rbae070_supplementary_data.docx]

**Supplementary Information**

## In situ co-deposition synthesis for collagen-Astragalus polysaccharide composite with intrafibrillar mineralization as potential biomimetic-bone repair materials

Han Li^1,2^, Ziying Guan^1,2^, Liren Wei^1,2^, Jian Lu^1,2^, Yanfei Tan^1,2^, Qingrong Wei^1,2,*^

1 National Engineering Research Center for Biomaterials (NERCB), Sichuan University, Chengdu 610065, P. R. China

2 College of Biomedical Engineering, Sichuan University, Chengdu 610065, P. R. China

*Correspondence address. E-mail: qingrongwei@scu.edu.cn

## Method

### 1.1 The isolation method of BMSC cells

Following deep anesthesia and euthanasia of the SD rats, bilateral separation of the femur and tibia was performed using sterile instruments. The bone marrow cavities of the femur and tibia were rinsed repeatedly with α-MEM medium containing 10% fetal bovine serum and collected in centrifuge tubes. The supernatant was removed by centrifugation and transferred to culture dishes, which were incubated at 37 °C in a 5% CO_2_ incubator. After three days of incubation, the medium was replaced with fresh α-MEM to remove non-adherent cells. On the seventh day of incubation, the cells in the culture dish were observed to have fused to approximately 90% for cell progeny culture.

## Results

**
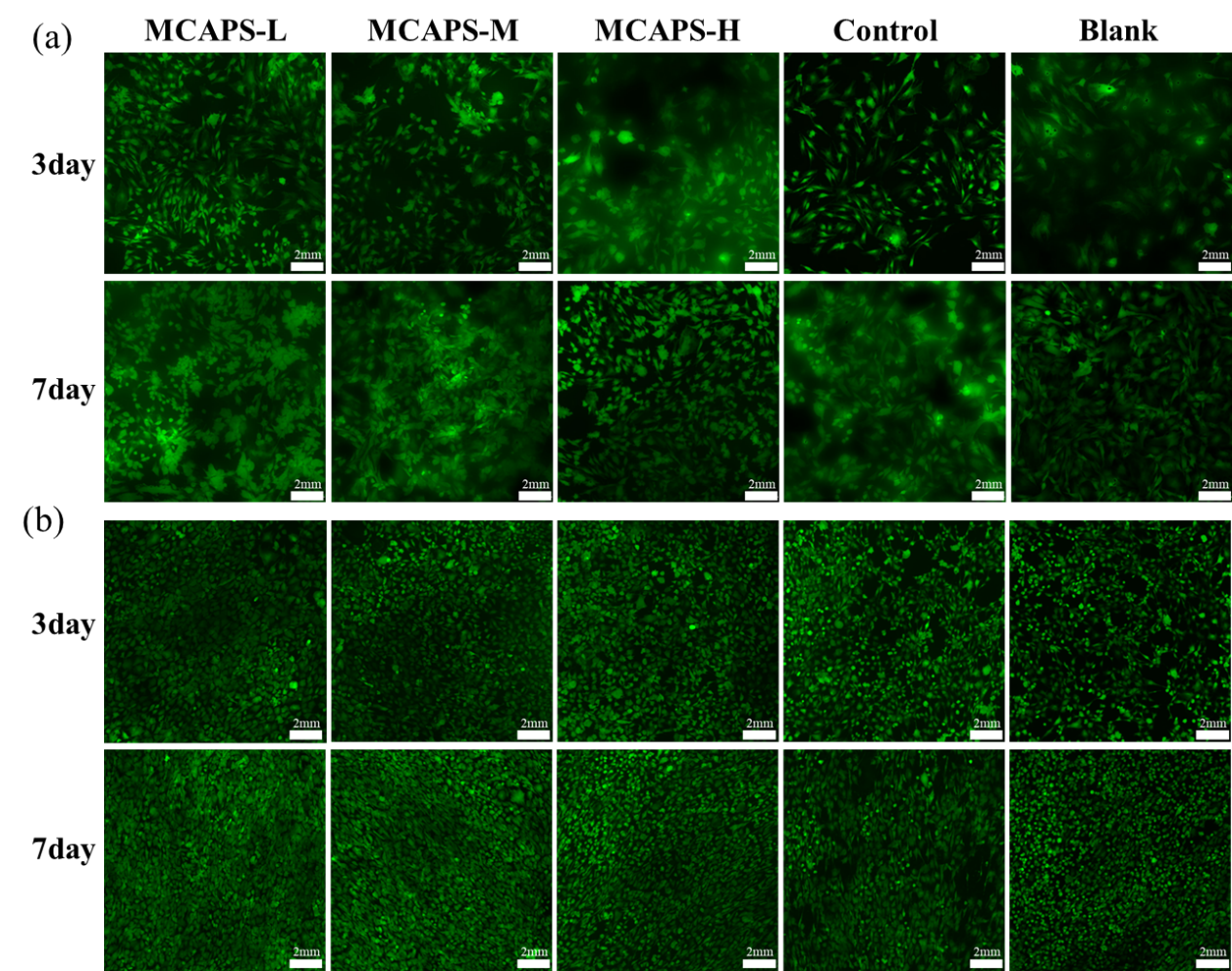
**

**Figure. S1.** The live/dead staining of BMSC (a) and MC3T3-E1 (b) at high magnification.

**
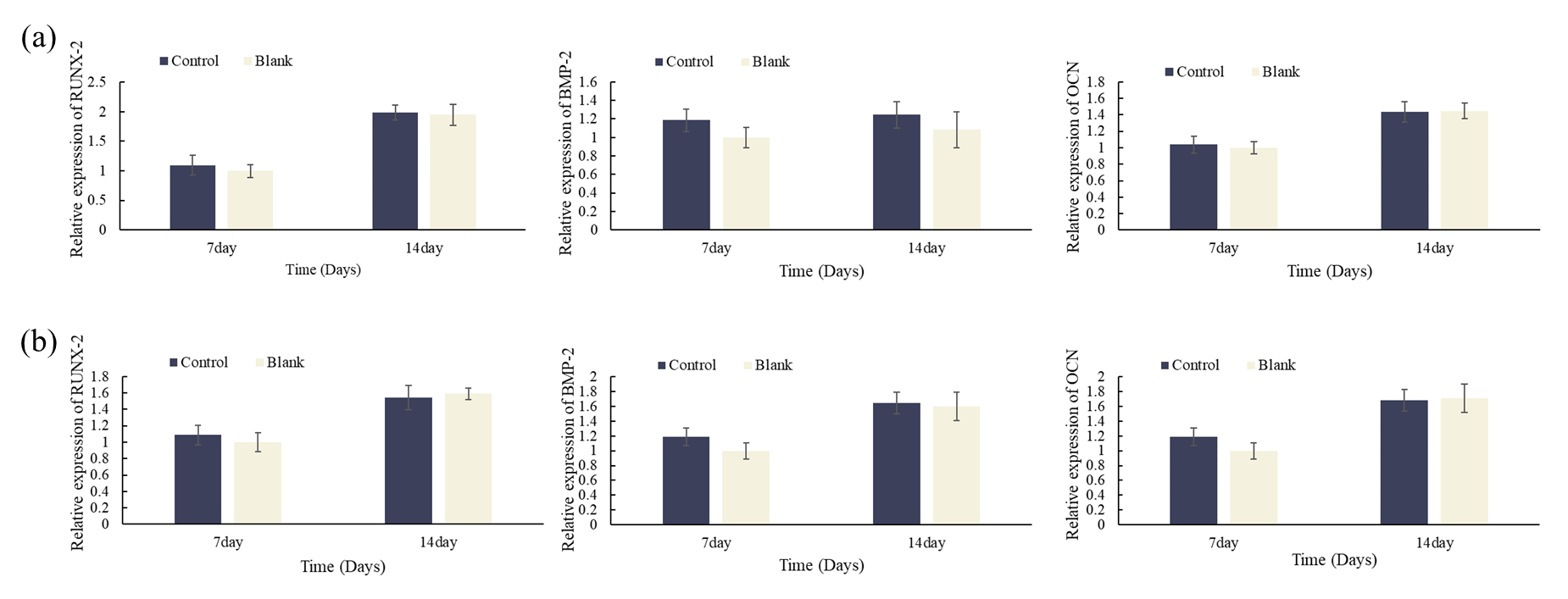
**

**Figure. S2.** The relative mRNA expression of Runx-2, BMP-2, and OCN in in the Control and Blank groups of BMSC (a) and MC3T3-E1 (b) at day 7 and 14.
